# Role of bacterial efflux pumps in antibiotic resistance, virulence, and strategies to discover novel efflux pump inhibitors

**DOI:** 10.1099/mic.0.001333

**Published:** 2023-05-24

**Authors:** Amit Gaurav, Perwez Bakht, Mahak Saini, Shivam Pandey, Ranjana Pathania

**Affiliations:** ^1^​ Department of Biosciences and Bioengineering, Indian Institute of Technology Roorkee, Roorkee, Uttarakhand, India

**Keywords:** efflux pump, EPI, AcrAB-TolC, antibacterial adjuvants, Antibiotic resistance, machine learning

## Abstract

The problem of antibiotic resistance among pathogenic bacteria has reached a crisis level. The treatment options against infections caused by multiple drug-resistant bacteria are shrinking gradually. The current pace of the discovery of new antibacterial entities is lagging behind the rate of development of new resistance. Efflux pumps play a central role in making a bacterium resistant to multiple antibiotics due to their ability to expel a wide range of structurally diverse compounds. Besides providing an escape from antibacterial compounds, efflux pumps are also involved in bacterial stress response, virulence, biofilm formation, and altering host physiology. Efflux pumps are unique yet challenging targets for the discovery of novel efflux pump inhibitors (EPIs). EPIs could help rejuvenate our currently dried pipeline of antibacterial drug discovery. The current article highlights the recent developments in the field of efflux pumps, challenges faced during the development of EPIs and potential approaches for their development. Additionally, this review highlights the utility of resources such as natural products and machine learning to expand our EPIs arsenal using these latest technologies.

## Introduction

**Fig. 1.**
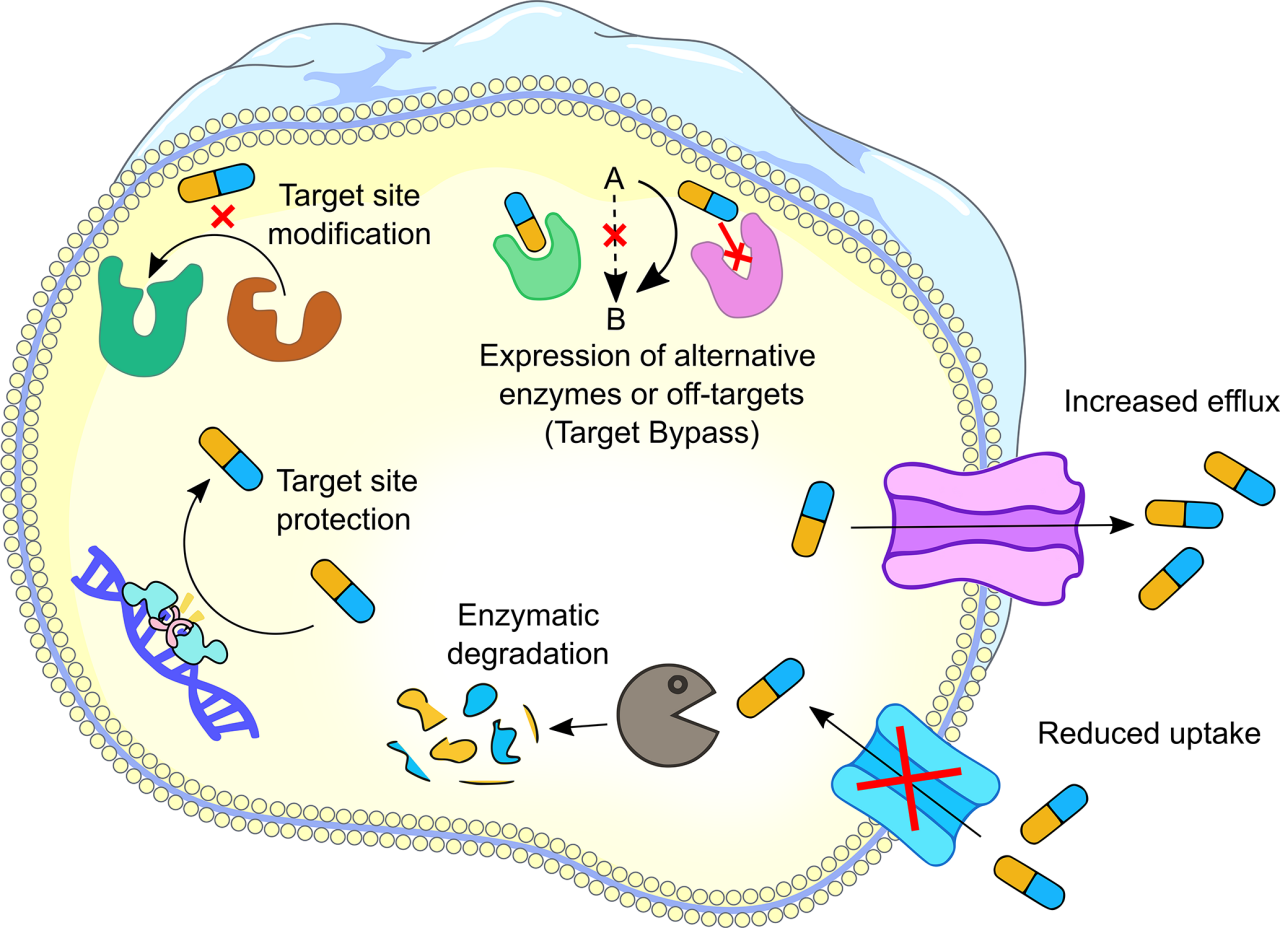
Schematic representation of antibiotic resistance mechanisms. Six different resistance mechanisms are found in bacteria. (**a**) Increased efflux by efflux systems; (**b**) reduced uptake due to change in membrane permeability; (**c**) enzymatic degradation; (**d**) target site protection; (**e**) target site modification and (**f**) expression of alternative enzymes or off-target sites.

The discovery of antibiotics in the mid-twentieth century transformed medical sciences and consequently enhanced life expectancy across the globe [1]. Antibiotics provide effective infection management, which enables us to deal with complex medical procedures such as surgeries, organ transplants, cancer therapy, and many more [2]. The joy of this antibiotic era, however, did not last long due to the simultaneous evolution of antimicrobial resistance (AMR) [3]. In the current era, antibiotic-resistant bacteria are a global threat to health and livelihoods. Though antibiotic resistance predates the use of antibiotics clinically, inappropriate and misuse of antibiotics has led to the evolution of new AMR mechanisms in pathogenic bacteria [4–7]. The emergence and global spread of AMR jeopardize the ability to treat infectious diseases and raises healthcare costs. The 2017 report of the World Health Organization on antibiotic resistance priority pathogens also highlights the importance of focusing research on these deadly microbes [8]. Antibiotic resistance in bacteria can be developed through six major mechanisms; (a) limiting the uptake of the antibiotic by altering the cellular permeability, (b) modifying the antibiotic target site, (c) target site protection, (d) enzymatic inactivating of the antibiotic, (e) active antibiotic efflux pumps, and (f) target bypass (Fig. 1) [9].

**Fig. 2.**
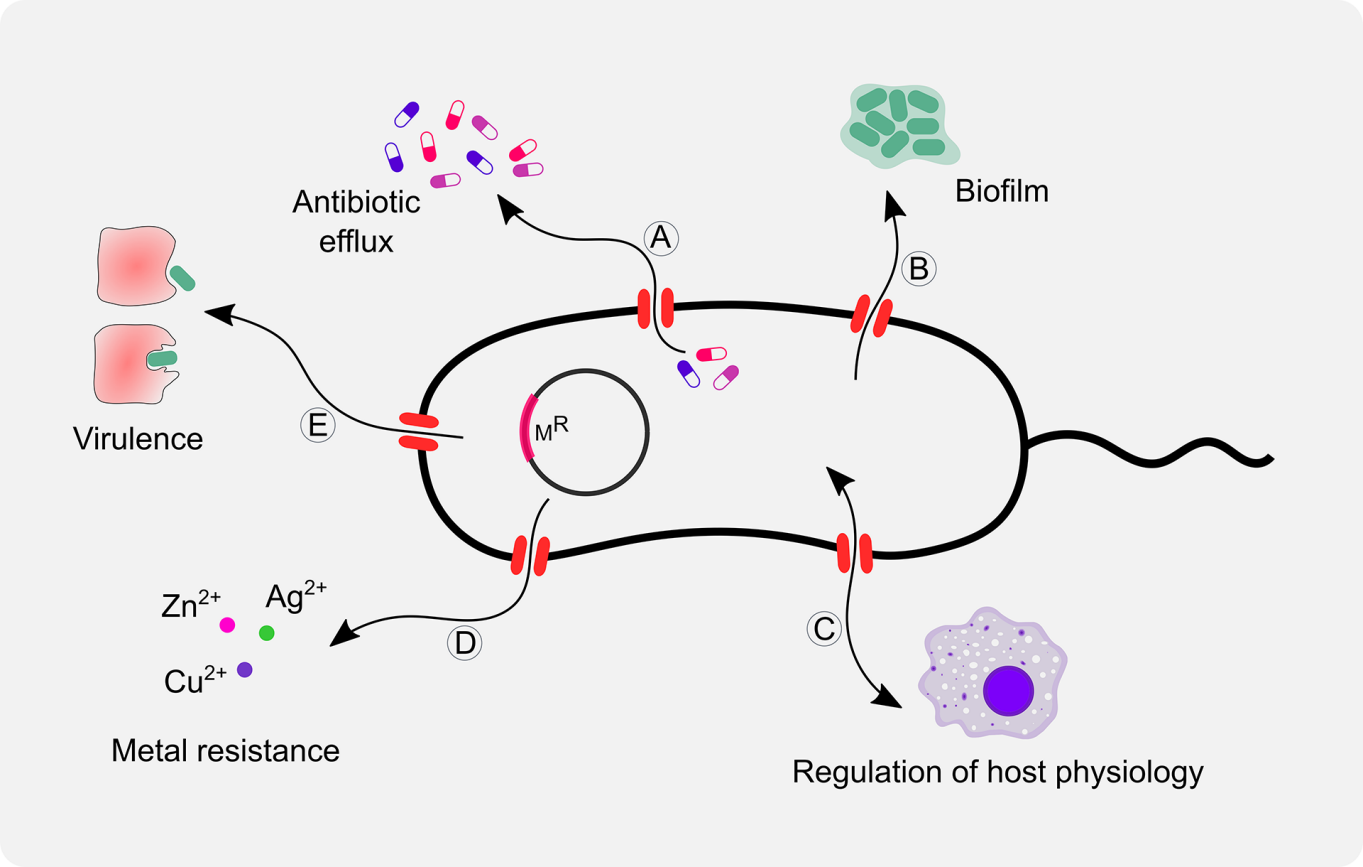
Schematic representation of biological functions of bacterial efflux pumps. Efflux pumps play an important role in (**a**) efflux of antibiotics, (**b**) biofilm formation, (**c**) regulation of host physiology, (**d**) metal resistance, and (**e**) virulence.

**Fig. 3.**
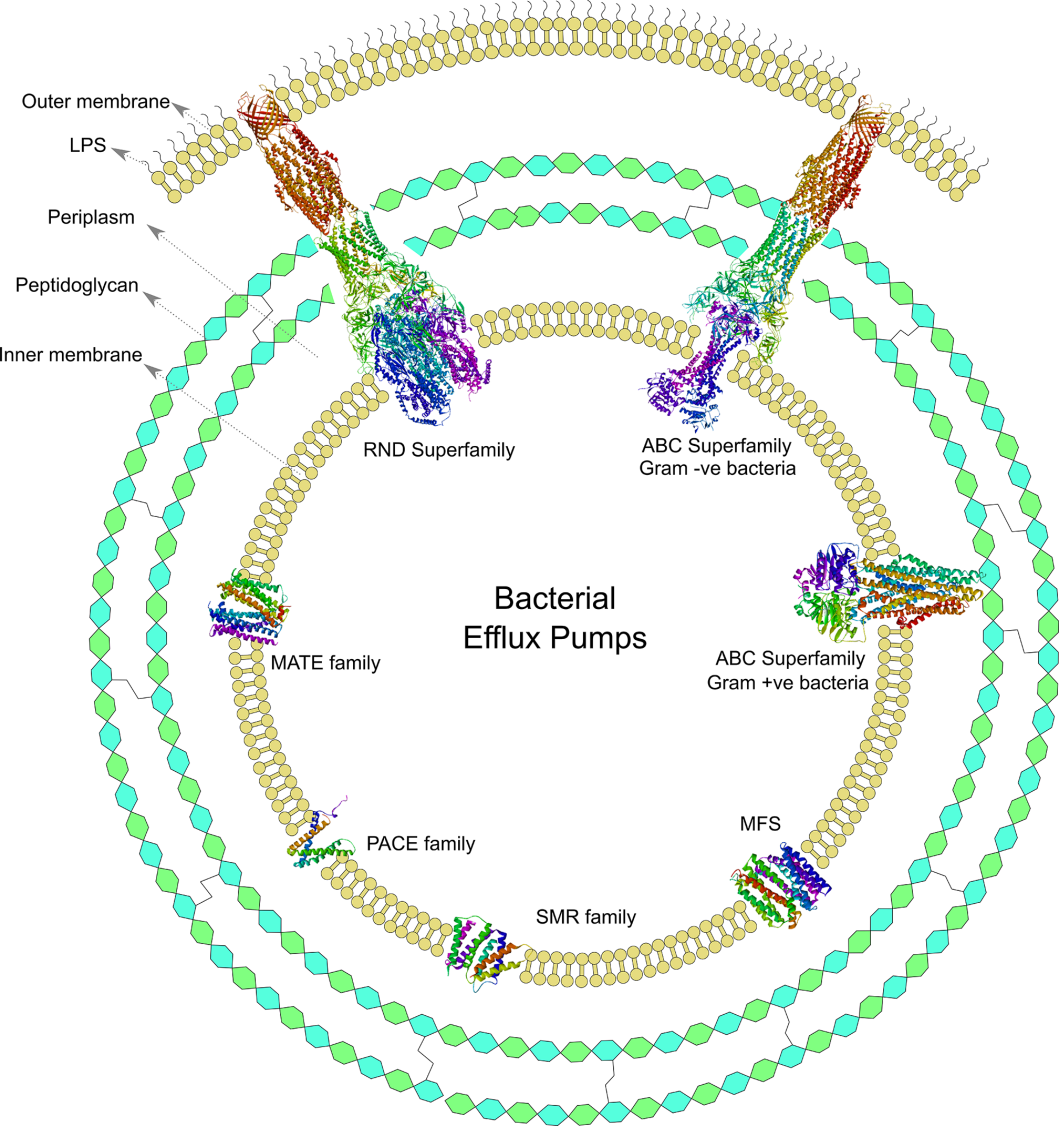
Schematic representation of bacterial efflux pumps. All bacterial efflux pumps are located on the inner membrane. Gram-negative bacteria have three components in their cell envelope, i.e. outer membrane, peptidoglycan layer, and inner membrane. Gram-positive bacteria have only two components in their cell envelope, i.e. peptidoglycan layer and inner membrane. Representative structures from each family (superfamily) have been presented here. Currently, six types of efflux pump families have been identified in bacteria, i.e. ATP-binding cassette (ABC) superfamily, major facilitator superfamily (MFS), small multidrug resistance (SMR) family, proteobacterial antimicrobial compound efflux (PACE) family, multidrug and toxin extrusion (MATE) family, and resistance-nodulation-cell division (RND) superfamily. The following PDB (Protein Data Bank) identifiers were used for depicting 3D structures; 2HYD for the ABC superfamily (Gram-positive); 3VVN for the MATE family; 4ZOW for MFS; 5NIK for the ABC superfamily (Gram-negative); 5V5S for RND superfamily; and 6WK9 for SMR family. Three dimensional structure of the PACE family (GeneBank ID: A1S_2063) was predicted using the ColabFold algorithm [119]. Then 3D structures were rendered using BIOVIA Discovery Studio Visualizer (Dassault Systems, France).

Antibiotic efflux is one of the most common mechanisms of resistance among a wide range of pathogenic bacteria [10, 11]. Efflux pumps are transport proteins localized in the cytoplasmic membrane of bacteria that actively translocate the chemical across the membrane. Efflux pumps are involved in the regulation of the internal environment by extruding out the toxic substances, quorum sensing molecules (autoinducers), biofilm formation molecules, and virulence factors of the bacteria (Fig. 2) [12]. Efflux pumps can confer heavy metal resistance by exporting metal ions such as Ag^2+^, Cu^2+^, Co^2+^, Zn^2+^, Cd^2+^ and Ni^2+^. In Gram-negative bacteria, efflux pumps help to reduce not only the cytoplasmic concentration of heavy metal ions but also the periplasmic concentration; since periplasmic metal ions can re-enter the cytoplasm, multiple efflux pumps work together to evade heavy metal toxicity [13–15]. Efflux pumps are categorized as primary and secondary on the basis of the source of energy they utilize to pump out the substrates. The efflux pumps that drive energy from the hydrolysis of ATP to translocate the substrates across the membrane are defined as the primary efflux pumps, whereas those which draw energy from electrochemical gradients formed by protons or ions (proton motive force) are defined as the secondary efflux pumps. Till now, six major families of efflux pumps have been found in bacteria, namely, ATP-binding cassette (ABC) superfamily, major facilitator superfamily (MFS), multidrug and toxic compound extrusion (MATE), resistance nodulation cell division (RND) family, small multidrug resistance (SMR) family and proteobacterial antimicrobial compound efflux (PACE) family (Fig. 3) [16]. Even though there are various types of efflux pumps, substrate redundancy exists among all classes of efflux pumps, such that one antibiotic can be exported by several different efflux pumps and a single efflux pump can export structurally and chemically diverse substrates [17]. Efflux pumps play an essential role in different stress environments for bacteria; thus, they can be a promising target for developing new inhibitors to rejuvenate obsolete existing antibiotics. However, due to the substrate redundancy of efflux pumps, the clinical success of efflux pump inhibitors is uncertain. This challenge can be addressed by discovering novel broad-spectrum efflux pump inhibitors.

The current review summarizes recent advancements in our understanding of the role of efflux pumps in bacterial physiology. Furthermore, this review will focus on different strategies for the development of efflux pump inhibitors, such as chemoinformatics and machine learning.

## Types of bacterial efflux systems

### ATP-binding cassette (ABC) superfamily

The ABC superfamily is a primary efflux pump family and it draws energy from the active hydrolysis of ATP to translocate a wide range of solutes, including drugs, lipids, and sterols across the membrane [18, 19]. All ABC families share a basic common architecture of two membrane-integral part domains that transverse the membrane six times each (12 transmembrane domains [TMDs]) and two nucleotide binding domains (NBDs) where the TMDs are involved in substrate-binding and NBD bind and hydrolyse ATP to make the transport cycle work [16, 20, 21]. ABC extrusion systems are divided into ‘full’ or ‘half’ transporters. In full transporters, a single polypeptide encodes two NBDs and two TMDs. In half transporters, a single polypeptide encodes for one NBD and one TMD [18, 22]. The ABC family functions according to an 'alternate access' mechanism between the inward-facing (IF) and the outward-facing (OF) to extrude substrates across the membrane, but the extent of physical separation between the two NBDs in the inward-facing (IF) and the outward-facing (OF) states is still unsettled [16]. In addition, a recent study reported on the physical segregation between two NBDs and found that NBD segregation exclusively decreases from inward to outward facing state, whereas NBD segregation seems larger when ADP is bound to it [23]. ABC transporters found in human pathogenic bacteria contribute to virulence, pathogenesis, and multidrug resistance via various mechanisms. ABC transporters have dual functionality, i.e. they can act as an importer or an exporter. ABC importers aid virulence by acquiring essential nutrients such as peptides, vitamins, amino acids, transition metals, and osmoprotectants. ABC transporters contribute to virulence by exporting essential molecules involved in glycoconjugate biosynthesis such as lipopolysaccharides and capsular polysaccharides, as well as by exporting xenobiotics [24]. In *Salmonella enterica* serovar Typhimurium, MacAB an efflux pump belonging to the ABC family is shown to aid its survival when exposed to oxidative stress inside the macrophages [25]. Transition metals play critical role in pathogenesis of several human pathogens [26]. ABC family efflux pumps are reported to be involved in export of transition metals in major human pathogens like *Pseudomonas aeruginosa *, *Listeria monocytogenes *, and *Mycobacterium tuberculosis* [27–29]. The diverse functionality of ABC transporters makes them an attractive target for antibacterial drug development.

### Resistance nodulation cell division (RND) superfamily

The RND superfamily mainly consists of twelve transmembrane helices that are separated by two large loops to form asymmetric trimers and the outer loops contain binding sites for exported ligands, while the transmembrane domain mainly functions as a channel for protons to utilise energy for substrates translocation [30]. Though 12 helices are signature of RND efflux pumps, sometimes more than 12 helices have been reported, for example, SecYEG of *Escherichia coli* has 15 transmembrane helices [31]. RND efflux pumps are known to work as a trimer (for example HAE [hydrophobic and amphiphilic efflux]-subfamily efflux pumps, including AcrB of *E. coli *, MexB of *P. aeruginosa *, MtrD of *Neisseria gonorrhoeae *, CmeB of *Campylobacter jejuni *, and HME [heavy metal efflux]-subfamily proteins, such as CusA and ZneA), although recent studies suggest that these transporters may be either dimers (HpnN membrane protein of *Burkholderia multivorans *) or monomers (MmpL3 transporter of *Mycobacterium smegmatis *) [16, 32]. The RND superfamily is the most potent efflux pump family mediating antibiotic resistance in Gram-negative bacteria. The pump is composed of an outer membrane factor (OMF), a periplasmic adapter protein (PAP) and an inner membrane RND-transporter, and thus also called tripartite resistance-nodulation-division (RND) efflux pumps [33–35]. Furthermore, the PAP is a complex protein comprised of four domains: helical, lipoyl, barrel, and membrane-proximal domains (MPD). It links inner membrane transporters and OMFs to form continuous conduits across the membrane [36, 37]. One of the most common enteric pathogens that affects both humans and animals is *S. enterica* serovar Typhimurium. Consumption of contaminated food and water is the main source of bacteria reaching the intestinal epithelial and it causes gastrointestinal disease [38]. Previous reports on *S. enterica* have described that there are four three dimensional (3D) sites present in the promoter region of PAP sequence which are involved in the structural foundation for PAP-RND interaction. According to structural studies, these four 3D sites correspond to nine distinct linear binding sequences known as binding boxes [37]. Additionally, the aforementioned study identified critical conserved residues within the binding boxes responsible for RND efflux pump functionality and further refined the exact residues of box4 (T271 and F292), box1 (R59) and box9 (K366) critical for efflux function [37, 39]. The mutation of the identified conserved residues of each binding box completely destabilises an RND-based tripartite efflux pump, the AcrABZ-TolC assembly. These newly identified residues can be employed as novel therapeutic targets for the development of efflux pump inhibitors to combat antimicrobial resistance [37, 39]. RND superfamilies are known for their role in virulence as well as resistance. The capacity for bacterial adhesion and invasion of host cells is crucial for effective colonisation and infection [40]. A recent study found a significant reduction in adhesion and invasion in efflux pump mutants of *E. coli* in comparison to parental strains, which is incongruent with a previous report where lack of AcrB in *S. enterica* reduced adhesion and invasion, supporting a relevant role of the RND efflux pump in bacterial virulence [41, 42].

### Major facilitator superfamily (MFS)

The MFS family is the largest known superfamily of secondary active transporters found ubiquitously in bacteria, archaea, and eukaryotes. It includes members that are solute uniport (movement of solute independent of ions), solute/cation symport (movement of solute and ion in the same direction), solute/cation antiport (movement of ion and solute in opposite directions) and/or solute/solute antiport with inwardly and/or outwardly directed polarity [43, 44]. Both symporters and antiporters use energy from the proton motive force (PMF) to move substrates across the membrane [45]. The majority of this family functions as a monomeric unit and possesses 12 to 14 TMHs which are organized into two domains, each as a bundle of six helices [46]. Surprisingly, it is still unclear how MFS transporters are allosterically regulated down to the molecular level, but with improvements in single-particle cryo-EM, and other techniques such as fluorescence resonance energy transfer (FRET), nuclear magnetic resonance (NMR) and electron paramagnetic resonance (EPR) spectroscopy, this can expand structural data and reveal novel, conceptual insights into the MFS transporters [47, 48]. MFS transporters play important roles in host–pathogen communication, especially in adhesion, invasion, intracellular survival, and biofilm formation. Inhibiting the activity of MDR transporters is a promising strategy to combat drug resistance and reduce virulence of pathogens, e.g. inactivation of *Acinetobacter baumannii* MFS efflux pump, AbaF, reduces bacterial virulence in a *Caenorhabditis elegans* model [49, 50]. *N. gonorrhoeae* is a strict human pathogen that causes gonorrhoea, a sexually transmitted disease. FarAB, an MFS pump in *N. gonorrhoeae,* mediates resistance to long-chained fatty acids like oleic, linoleic, and palmitic acids [51]. *L. monocytogenes* is a foodborne pathogen that can break the intestinal barrier, and rapidly spread to liver and spleen. Two MFS pumps, MdrM and MdrT, play important roles in pathophysiology of *L. monocytogenes *. Both MdrM and MdrT are involved in bile resistance and modulate the cytosolic surveillance pathway of innate immunity, which promotes bacterial spread and tissue invasion. MdrM actively controls cytosolic bacteria’s ability to induce IFN-β expression [52].

### Multidrug and toxic compound extrusion (MATE) family

The MATE family was first reported 24 years ago and was believed to be closely related to the MFS family, although computational studies and structural characterization show that both the MATE and MFS families differ in their sequence and topology [53–55]. MATE transporters are classified into the multidrug/oligosaccharide-lipid/polysaccharide (MOP) flippase superfamily and further segregated into NorM, DNA damage-inducible protein F (DinF) and eukaryotic superfamilies based on their amino acid sequence similarity [16, 56]. Several crystal structures of the MATE superfamily transporters have shown that they consist of 12 transmembrane helices with an N-lobe and a C-lobe belonging to intramolecular pseudo twofold symmetry, with an axis perpendicular to the membrane plane [55]. Despite structural similarities, the transport mechanisms of a few MATE superfamily members have been observed to differ, for example, NorM-Vc from *Vibrio cholerae* and NorM-PS from *Pseudomonas stutzeri *. NorM-VC and NorM-PS have comparable structural properties, however NorM-PS is driven by H^+^ electrochemical gradients, while NorM-VC has been found to be coupled to both Na^+^ and H^+^ [57]. To translocate cationic substrates across the membrane, the MATE superfamily functions as a secondary antiporter (influx of H^+^ or Na^+^) and uses a rocker-switch alternating access mechanism, switching between substrate bound outward facing or ion-bound inward-facing conformations [53]. Ethidium bromide, berberine, acriflavine, norfloxacin and tetraphenylphosphonium are some of the cationic substrates that MATE transporters extrude and reduce susceptibility to these drugs in bacteria [58]. Bacteria are also prone to developing resistance following changes in the expression level of the MATE superfamily, for example overexpression of MepA in *Staphylococcus aureus* can lead to resistance against tigecycline, which is used to treat methicillin and vancomycin-resistant *S. aureus* infections [58]. The MATE superfamily efflux (*A1S_3371*) pump has been reported to contribute to *A. baumannii* ATCC 17978 virulence [59].

### Small multidrug resistance (SMR) family

The SMR family is composed of small (12 kDa) integral inner membrane proteins containing only four transmembrane α-helices, which confer resistance to a variety of quaternary ammonium compounds and other lipophilic cations in archaea and bacteria [60]. Despite being small in size, the SMR family functions as homodimers or heterodimers. The overall mechanism of SMR family for transport is an exchange between the substrate and a proton (antiport) [61]. A wide range of SMR family studies have shown that multidrug resistance is driven by the proton motive force and that the conserved amino acid glutamic acid is an important residue that contributes in expelling cationic drugs [62, 63]. Several SMR proteins have been identified in bacterial pathogens and resistance has been found against clinically used antibiotics such as β-lactams, aminoglycosides, inhibitors of dihydrofolate, and various antiseptics [64]. *M. tuberculosis* is a pathogenic bacterium and the causative agent of tuberculosis that contains the *mmr* gene which encodes the Mmr protein. Overexpression of Mmr protein reduces the bacterial susceptibility to ethidium bromide, quaternary ammonium compounds, and a few antibiotics such as kanamycin and amikacin [65, 66]. As far as we know, little evidence has been shown for the contribution of MATE transporters to bacterial virulence in human pathogens.

### Proteobacterial antimicrobial compound efflux (PACE) family

The PACE family proteins, with AceI from *A. baumannii* as the prototype, are a recently discovered family of bacterial drug efflux transport proteins that are encoded by genes in the bacterial core genome rather than by mobile genetic elements, which suggests that they provide some important function [67]. In addition, AceI exhibits a wide range of resistance against structurally diverse antimicrobial compounds and biosynthetic biocides (e.g. benzalkonium, diqualinium, acriflavin, proflavin, and chlorhexidine) [68]. An alignment of 47 different PACE family proteins from various bacterial species revealed that four amino acid residues, glutamic acid, asparagine, alanine, and aspartic acid, appeared to be conserved across the family. Glutamic acid is found in transmembrane helix one, asparagine is found in transmembrane helix two, alanine is located at the boundary of the periplasmic membrane of transmembrane helix four, and aspartic acid is located at the boundary of the cytoplasmic membrane of transmembrane helix four [68]. AceI of *A. baumannii* is the prototype PACE family member involved in the transport of widely used antiseptic – chlorhexidine. AceI’s glutamic acid (E15) was discovered to be fully conserved and responsible for proton binding. In a recent study, E15 was found to play an important role in the dimerization of AceI proteins in solution, where monomeric and dimeric forms of AceI proteins exist in a dynamic equilibrium and the equilibrium state is modulated by pH, cardiolipin and chlorhexidine binding. Further, mutation of this glutamic acid into glutamine (E15Q) results in a significantly different pH response than the wild-type AceI protein [69]. The C-terminus of the AceI protein was found to be highly conserved as compared to the N-terminus, which suggests that it plays an important role in core function and, on the other hand, the N-terminus plays a major role in substrate recognition. Short-chain diamines (such as cadaverine and putrescine) that are known for their important roles in metabolism, transcription regulation, and protein expression were found to be physiological substrates of PACE family transporters [70]. PACE transporters translocate their substrates using energy generated through the electrochemical proton gradient across the membrane, for instance the PACE transporter PA2880 from *P. aeruginosa* mediates chlorhexidine efflux by employing the proton motive force [71]. The lack of genes encoding proteins of the PACE family in the *E. coli* chromosome also suggests that these genes were lost early in the divergence from the Gammaproteobacteria [16].

## Strategies to discover novel efflux pump inhibitors that can revive the activity of ineffective antibiotics

Antibiotic resistance has grown parallelly whenever new antibiotics have been introduced to the market [6, 7]. Although some antibiotic resistance determinants were present before the introduction of a particular antibiotic in the market; high usage of antibiotics throughout the world exerts a selection pressure on bacteria and that can contribute to rise in antibiotic resistance [4, 5, 72–74]. Efflux pumps are major determinants of antibiotic resistance and hence identification of chemical moieties capable of reversing the effect of efflux pumps is needed. Several strategies have been employed to discover efflux pump inhibitors (EPIs) in the past. This section will focus on previously used strategies as well as the scope of introducing emerging approaches like machine learning in discovery of EPIs.

### Plant-derived secondary metabolites – a good starting point for EPIs

Combinatorial chemistry has changed how we think of developing a new chemical entity (NCE) however, plant-based natural products have always been a source of human medicines. Natural products have been the single most productive source for lead identification and further development of drugs. Plant metabolites have been actively used in drug development for many therapeutic areas like cancer, neurological, cardiovascular, skin, gastrointestinal, inflammation, and metabolic disorders [75]. Additionally, many plant metabolites like terpenoids, phenolic compounds, and alkaloids show moderate antibacterial activity [76]. The direct antibacterial effect of these secondary metabolites is due to their ability to hamper bacterial protein synthesis, DNA synthesis, and RNA synthesis; however, their effect on the cell envelope is quite prominent [77]. Plant secondary metabolites can cause severe bacterial cell envelope stress and that triggers a cascade of events such as cell wall damage and the leakage of cytoplasmic constituents, metabolites and ions [76]. Although many intensive screenings have been done to discover potent plant secondary metabolites as antibacterial agents, however, there is a scarcity of literature describing the screening of plant secondary metabolites as efflux pump inhibitors [78, 79]. Targeted screening of plant secondary metabolites as EPIs is an important and currently overlooked strategy. Out of approximately 250 000 higher plants worldwide, only about 14–28 % have been investigated for a medical purpose [79]. Thus, this inadequately explored wealth of medicinal plants could be our ‘goldmine’ for the discovery of novel and effective efflux pump inhibitors. In the quest of finding novel antimicrobial entities during an antimicrobial screening of medicinal plants, we might have missed many potential and potent EPIs because priority is usually given to plant metabolites and their derivatives showing evident and significant growth inhibition of test pathogens [80]. Here, we propose a new perspective that could help identify novel EPIs from fractions of plant metabolites. An ideal EPI should not possess inherent antibacterial activity, as any chemical entity exerting direct stress on bacterial physiology will suffer the same consequence as of any antibiotics, i.e. the rapid development of resistance and we have faced such outcomes with many antibiotics in the past [74].

The rise in the incidence of MDR phenotypes among pathogenic bacteria has consequently forced us to use combinations of antibiotics. The depth of this crisis level can be understood by the fact that more than half (55%) of the combination drugs approved by the Food and Drug Administration (FDA) are used to treat infections [81]. However, currently, there are no approved EPIs for clinical usage. There might be several factors for such a void; the first is the diversity of efflux pumps available to any pathogen. Both Gram-positive and Gram-negative pathogens have multiple functional efflux pumps. Additionally, broad substrate specificity is a hallmark of almost all efflux pumps; it becomes even more complicated to develop EPIs. Surprisingly, plants have developed multiple arsenals to tackle this problem. Most of the plant secondary metabolites do not have inherent antibacterial activity except a few with moderate antibacterial activity. Indeed, many of them act as antiherbivoral and other molecules required for interspecies competition [80, 82].

**Fig. 4.**
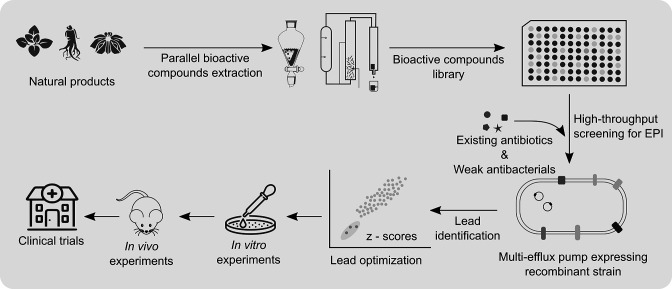
Schematic representation describing an approach to discover new efflux pump inhibitors (EPIs) from the natural products library. Screening of the natural products library could help identify novel EPIs or a group of related chemical moieties that act synergistically as a potent antibacterial agent. Screening for EPIs could be performed against recombinant strains expressing multiple efflux pumps to select a broad-spectrum EPI or collection of EPIs that can target different efflux pumps.

Interestingly, plants have developed many efflux pump inhibitors that act synergistically with other secondary metabolites like alkaloids and thus making a non-antibacterial secondary metabolite or moderately active secondary metabolite into a potent antibacterial [80]. For example, 5′-methoxyhydnocarpin-D (5′-MHC-D) is a flavonolignan produced by species like *Berberis fremontii, Berberis repens,* and *Berberis aquifolia* – berberine-producing native American plants. 5′-MHC-D has no antibacterial activity on its own. However, it acts as a very potent efflux pump inhibitor making berberine as effective as or even better than clinically used antibiotics against multiple drug-resistant *S. aureus* [83–85]. The overall idea is that the plants deploy a range of phytochemicals to potentiate a range of weak antimicrobials or narrow-spectrum antimicrobials to fight against all sets of invading bacterial pathogens. Hence, in order to harness the power of plant natural product diversity, we need to change our screening strategy. Since most of the plant diversity is unexplored for screening efflux pump inhibitors, there is an urgent need to look for new screening strategies that would yield novel efflux pump inhibitors. We need to collect plants from every corner of the world and a parallel bioactive compound extraction method could be followed. Previously reported weak and narrow-spectrum antibacterial phytochemicals as well as currently used antibiotics could be retested along with newly extracted phytochemicals. The major issue with the current efflux pump inhibitor screening is the lack of diversity; most of the screenings reported previously have been performed using recombinant strains expressing a single efflux pump like NorA, AcrAB‐TolC, MexAB-OprM or AbeM [84, 86–88]. The problem with the leads obtained from current screenings is that they inhibit only specific efflux pumps, however in clinical settings pathogens are equipped with efflux pumps from multiple classes [89–92]. These scenarios make discovering a clinically feasible efflux pump inhibitor an arduous task. In order to broaden the therapeutic range of an efflux pump inhibitor, screening against recombinant strains expressing multiple efflux pumps prevalent among clinical strains is essential (Fig. 4). Although the percentage of positive hits in such screening will reduce significantly due to stringent criteria, this will yield an enhanced confidence score. Structure-activity relationship studies of previously identified efflux pump inhibitors from different screening programmes may provide valuable information in this avenue [87, 88].

Conjugation of an efflux pump inhibitor to a weak antibacterial molecule is also a good strategy to start with [93, 94]. Berberine is a weak cationic antibacterial molecule that is prone to effluxed out by multiple efflux pumps in different pathogens. Surprisingly, conjugation of INF55 (an efflux pump inhibitor having no inherent antibacterial activity) with berberine yielded a potent antibacterial molecule with good *in vivo* efficacy against deadly enterococcal infection [94].

### Efflux pump inhibitors from the past

To date, several potent efflux pump inhibitors have been discovered. These efflux pump inhibitors belong to structurally diverse chemical classes like peptidomimetics [95], piperazines [96], pyridopyrimidines [97], and pyranopyrimidines [98]. MC-207,110 (Phe-Arg-β-naphthylamide or PAβN) is a broad-spectrum efflux pump inhibitor from peptidomimetics class that was identified using large scale screening of a chemical library [95]. Next, 1-(1-naphthylmethyl)-piperazine (NMP) is a potent efflux pump inhibitor from piperazines class specifically active against RND class efflux pumps [96]. D13-9001 is a potent and safe efflux pump inhibitor from pyridopyrimidines class which shows exceptional *in vivo* activity against *P. aeruginosa* infection [97]. MBX2319 is a recently discovered efflux pump inhibitor of AcrAB-TolC that can potentiate multiple antibiotics such as levofloxacin, piperacillin, and cefotaxime. Recently, two new efflux pump inhibitors (chlorpromazine and amitriptyline) have been identified using drug repurposing platforms. Both of these drugs have antipsychotic properties [99]. However, none of the above-mentioned molecules have been approved as efflux pump inhibitors for clinical usage basically due to *in vivo* cytotoxicity and other adverse effects [100].

### Machine learning-based discovery of novel efflux pump inhibitors

**Fig. 5.**
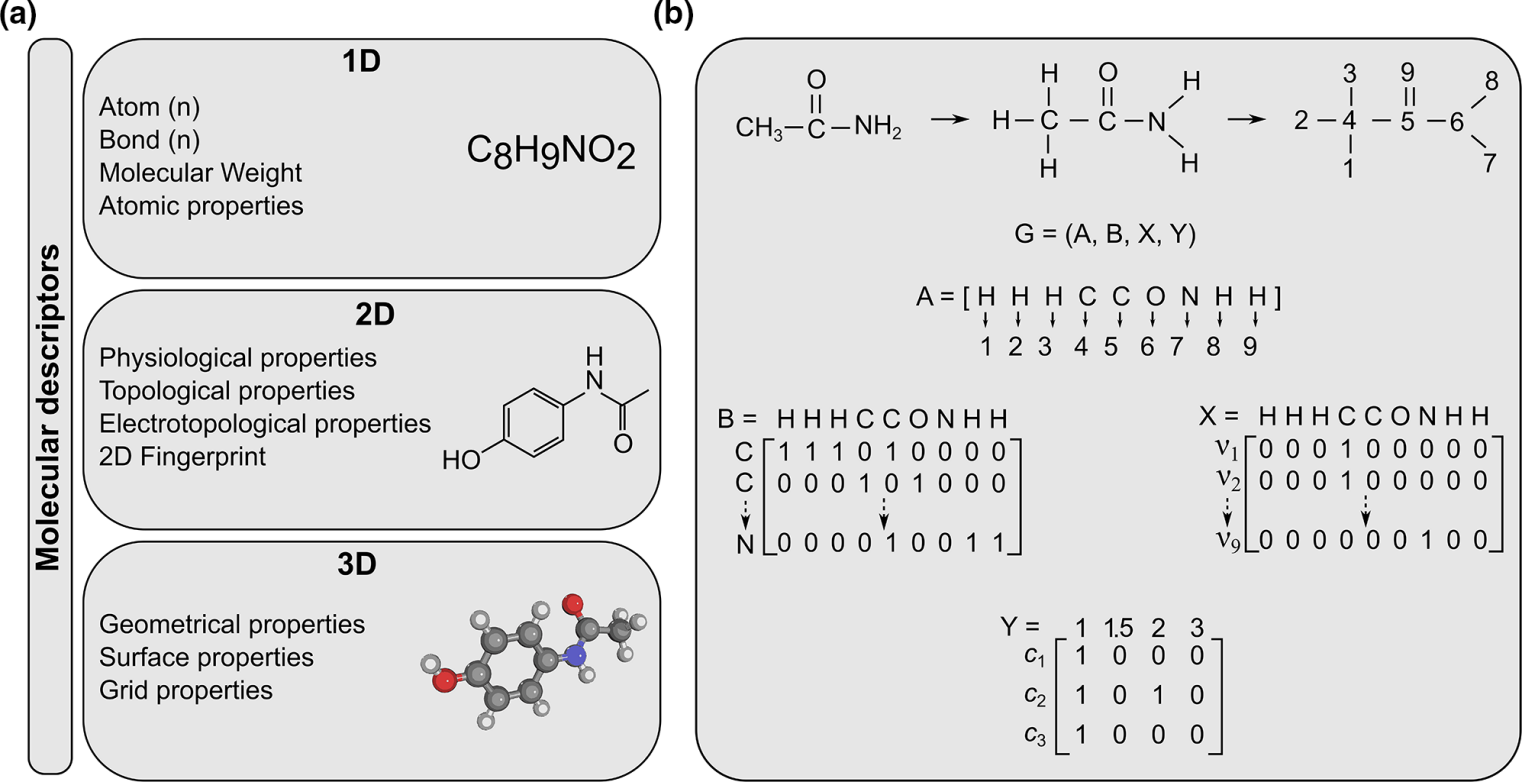
**(a)** Three categories of molecular descriptors; 1D (one-dimensional), 2D, and 3D descriptors. 1D descriptors depend on molecular formula; 2D descriptors contain 2D molecular fingerprints; 3D descriptors provide information about 3D geometric information of any molecule. (**b)** Schematic representation describing the process of making a graph convolution model of a molecule. G represents graph descriptors, A represents a set of atoms, B represents a set of bonds, and X and Y represent the atom content matrix.

The domain of chemoinformatics has changed over the past few years. There are several models for molecular property prediction of any chemical entity. Molecular fingerprinting is one of them and it is an essential cheminformatics tool for virtual screening and mapping chemical space. For small molecules, substructure fingerprints are the preferred technique, while for large molecules (e.g. antimicrobial peptides), atom-pair fingerprints are preferable. Still, there is no common method that achieves good performance on both classes of molecules [101]. Traditionally, molecules were represented by their molecular fingerprint vectors based on the presence or absence of functional groups in the molecule (Fig. 5a) [102]. The second strategy is to use molecular descriptors that are based on molecular properties and needs supervision by domain experts (Fig. 5b) [103]. These drawbacks and the limited accuracy of these models restrict their usage to small sets of molecules (a few thousand) [104]. However, recent innovations in neural network algorithms provide an opportunity to influence the paradigm of antibacterial drug discovery [104, 105]. Now any chemical entity (for example efflux pump inhibitor) can be denoted by a hybrid representation that contains both convolutions and molecular descriptors. This new algorithm outperforms any previous one by providing flexibility in learning a task around a fixed molecular descriptor (like a pharmacophore), simultaneously making convolutions around bonds rather than atoms. This last step also helps to reduce the total running time by averting unnecessary loops during the message-passing phase in the algorithm [105].

**Fig. 6.**
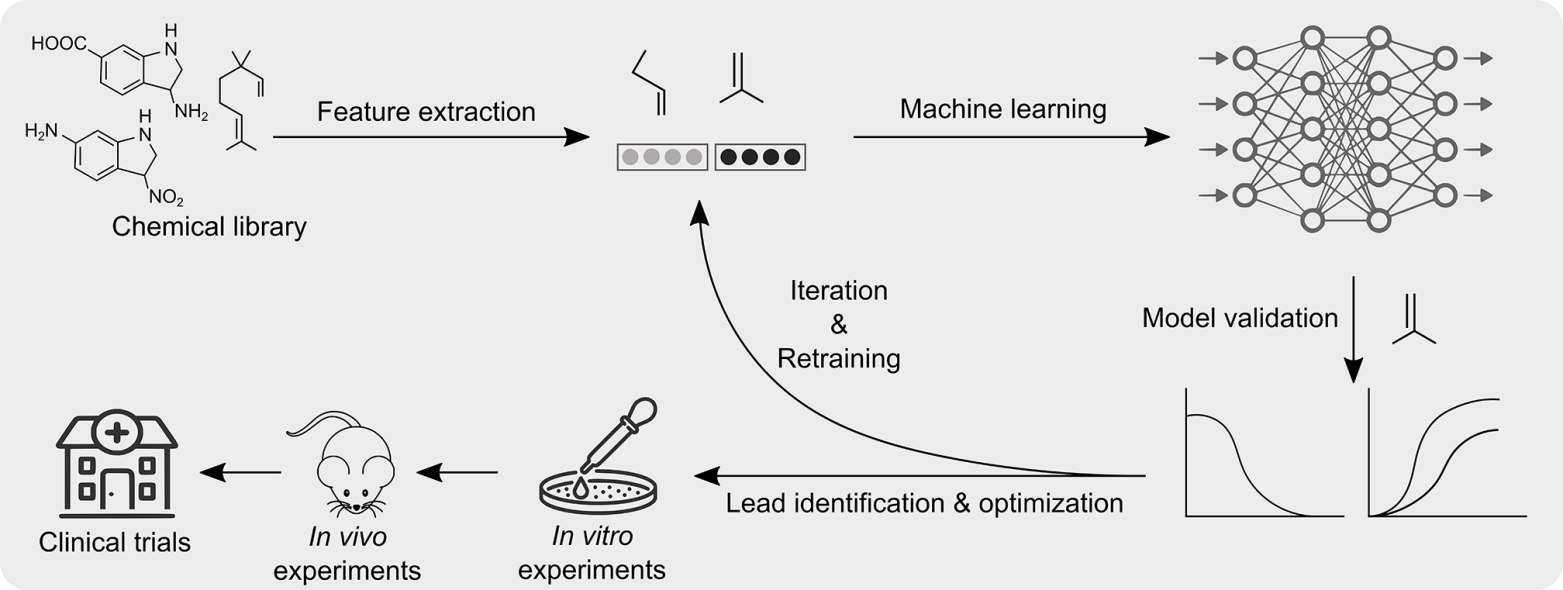
Schematic representation describing an approach to discover new efflux pump inhibitors (EPIs) using machine learning. The machine learning approach could help identify novel and robust EPIs using information already available for existing EPIs. Machine learning algorithms extract common feature among training data sets and implement them to find a lead among testing datasets.

Advancements in high-performance computing and parallel computing along with the availability of better computer hardware makes it a perfect time to implement machine learning for finding new antibacterial entities. Machine learning is a powerful tool that can be used to generate predictive as well as generative models which can help us to fight against bacterial infections. In recent times, several research groups have deployed machine learning approach to explore new antibacterials [104, 106–108]. Machine learning and computational approaches have helped to design novel antimicrobial peptides (AMPs) with enhanced efficacy and reduced toxicity [107]. Furthermore, the search for novel and cryptic antimicrobial peptides in the human genome and the human microbiome has yielded some interesting leads with excellent antibacterial activity [106, 108]. Recently, a deep learning approach has helped to discover an unusual class of antibiotic - Halicin, which has a broad-spectrum antibacterial activity [104]. One of the most important aspects of machine learning is the avoidance of the dereplication problem, wherein the same molecules are repeatedly discovered. The machine learning approach could filter out already reported antibacterial molecules. Recent advancements in the field of machine learning algorithms could definitely help us predict molecular properties of novel efflux pump inhibitors (Fig. 6). We can also capture vast chemical spaces *in silico* that are beyond the reach of the current experimental approach with far less associated running cost. Until now, search for novel efflux pump inhibitors using a machine learning approach has not been initiated (zero results on PubMed, searched on 24 November 2022). It is now more important than ever to screen for efflux pump inhibitors capable of restoring the effectiveness of ‘magic bullets’ using a deep learning approach. This can be achieved in three stages; first, the collection of molecular descriptors of positive hits from previously screened chemical libraries can be taken up [88, 95, 98, 109–112]. Second, training of a deep neural network(s) model to predict growth inhibition of test pathogen(s) using a combination of potential efflux pump inhibitor (positive hits from the previously screened chemical library) and test antibiotic can be taken up. Third, applying the best model to several discrete chemical libraries (with more than 100 million compounds) like ZINC15 and Maybridge to identify potential lead efflux pump inhibitors [113] (Fig. 6).

### Challenges and perspectives of bacterial efflux pump inhibitors

There are a few important concerns that need to be discussed before developing broad-spectrum efflux pump inhibitors. First, efflux pumps provide protection only in actively growing bacterial cells [114]. This could limit their potential usage against slow-growing or nongrowing pathogens where reduced membrane permeability plays a major role in maintaining low antibiotic concentration. Second, efflux pumps are not the one and only mechanism behind antibiotic resistance in bacteria. Third, all efflux pumps are not exclusive to the bacterial kingdom. Broad-spectrum ATP-dependent efflux pumps are also present in humans. MDR cancer cells often overexpress ATP-dependent efflux pumps to avoid toxicity caused by anti-cancer agents. A broad-spectrum efflux pump inhibitor may also target efflux pumps present on human cells and thus may show side-effects. Fourth, by virtue of their nature, efflux pump inhibitors will be used in combination with partner antibiotics. This poses an additional challenge to appropriately tailor the pharmacokinetic properties of both components of the combinations.

Thus many challenges are encountered on the path to conversion of a drug lead to a clinically valuable therapeutic agent. In this regard, an efflux pump inhibitor is no different from any other new chemical entity. However, what if we can hunt efflux pump inhibitors among existing drugs already used clinically for a different indication. This could significantly reduce time and resources for regulatory approval [115]. Similarly, many transporters are found in representatives of all kingdoms. However, homology between bacterial and human proteins is negligibly low and even if there are sequence similarities, conserved regions might be located in an integral inner membrane protein that does not participate in substrate specificity [116–118].

## Conclusion

Bacterial efflux systems play a crucial role in antibiotic resistance. They contribute to intrinsic as well as acquired antibiotic resistance in bacteria. The bacterial efflux system affects virtually all classes of antibiotics. Efflux pump inhibitors capable of restoring the effectiveness of available antibiotics are urgently needed. Untapped natural products can be a great resource for potential efflux pump inhibitors. Additionally, the machine learning approach can definitely help us to screen for new efflux pump inhibitors.
